# Changes in plasma IRAK-M in patients with prediabetes and its relationship with related metabolic indexes: a cross-sectional study

**DOI:** 10.1177/03000605221111275

**Published:** 2022-08-30

**Authors:** Xiaomin Xie, Guirong Bai, Li Zhang, Huili Liu, Dan Qiang, Ling Li

**Affiliations:** Department of Endocrinology, The First People’s Hospital of Yinchuan, Yinchuan, Ningxia, China

**Keywords:** Diagnostic factor, IL-1R-associated kinase, prediabetes, risk, type 2 diabetes, thioredoxin-interacting protein

## Abstract

**Objective:**

To investigate whether IL-1R-associated kinase (IRAK)-M is associated with prediabetes and type 2 diabetes (T2D).

**Methods:**

In this cross-sectional study, enrolled subjects were assigned to different groups according to their fasting plasma glucose (FPG) values. IRAK-M and metabolic parameters, including fasting insulin (FINS), glycosylated hemoglobin (HbA1c), homeostasis model assessment of insulin resistance (HOMA-IR) and beta-cell function (HOMA-β), and thioredoxin-interacting protein (TXNIP), were evaluated. The area under the receiver operating characteristic curve of IRAK-M and TXNIP for prediabetes and T2D was determined.

**Results:**

IRAK-M decreased significantly with increasing FPG levels. IRAK-M was negatively correlated with TXNIP, FPG, FINS, HbA1c, and HOMA-IR and positively correlated with HOMA-β. The diagnostic cutoff value of IRAK-M was 3.76 ng/mL for prediabetes and 3.45 ng/mL for T2D. After stratifying by IRAK-M (<3.76 and ≥3.76 ng/mL), patients with a higher TXNIP level showed a greater risk of prediabetes or T2D in the subgroup with low IRAK-M (<3.76 ng/mL).

**Conclusions:**

IRAK-M is independently and positively associated with prediabetes and T2D, while TXNIP is independently and negatively associated with prediabetes and T2D. IRAK-M and TXNIP serve as diagnostic factors for prediabetes.

## Introduction

Type 2 diabetes (T2D) is a metabolic disease associated with insulin resistance and pancreatic islet β-cell dysfunction. The development of T2D involves the chronic activation of inflammatory pathways.^1^ Toll-like receptors (TLRs), the most upstream pattern-recognition receptors in immune cells, recruit intracellular signaling molecules, including myeloid differentiation primary response gene 88 (MyD88), members of interleukin (IL)-1 receptor-associated kinases (IRAKs), and tumor necrosis factor (TNF) receptor-associated factor 6 (TRAF-6), leading to the activation of c-Jun N-terminal kinase, p38, and nuclear factor-kappa B (NF-κB)-dependent inflammatory responses.^1^ TLRs are activated by exogenous infectious ligands and respond to endogenous autoantigens released upon cell death or injury, inducing inflammation and leading to the development of autoimmune diseases and self-tissue damage.^1^

TLRs have been suggested to play a role in β-cell dysfunction and glucose homeostasis, priming the pathogenesis of T2D.^2,3^ The stimulation of TLRs triggers downstream pathways involving modulatory molecules and induces the production of the inflammatory cytokines IL-1β and IL-18.^4^ IL-1β levels are elevated upon exposure to high glucose, leading to sustained inflammation and diabetic cell damage/cell death.^5,6^ Increased serum levels of IL-18 were also observed in patients with T2D.^7^ The activation of TLR2 and TLR4 was shown to have negative effects on insulin resistance.^8^ A recent study using a T2D mouse model revealed that TLR4/MyD88/NF-κB signaling induces the production of IL-6, TNF-α, and monocyte chemoattractant protein, leading to the heart- and liver-related complications of T2DM.^9^ Inflammatory responses induced by TLR4 activation in human islets lead to β-cell failure.^10^ In an *in vivo* study, saturated fatty acids were shown to induce β-cell dysfunction by recruiting M1 macrophages to islets via the TLR4/MyD88 pathway.^11^ Macrophage-mediated TLR signaling may alter the islet cytokine secretome and increase immunoreactivity in islets, suggesting that TLR signaling provides a target for the treatment of islet inflammation in T2D.^12^ *In vivo*, β-cell death and its sensing via TLR2 may be an initial event for the stimulation of antigen-presenting cells and development of autoimmune diabetes.^13^ Additional studies revealed that the expression levels of TLR2 and TLR4 proteins in monocytes in the peripheral blood of patients with T2D are significantly higher than those in healthy people.^14–16^

IRAKs play important roles in TLR/IL-1R signaling. IRAK family members consisting of IRAK1, IRAK2, IRAK3 (IRAK-M), and IRAK4 mediate the activation of TLR and IL-1R signals and have a positive or negative regulatory effect on innate immunity, adaptive immunity, and inflammation.^17^ Current studies suggest a negative regulatory role of IRAK-M in TLR/IL-1R signaling through several mechanisms^14,15,18–21^ The expression of thioredoxin-interacting protein (TXNIP), which activates inflammatory pathways during the immune response, is also increased in patients with T2D.^22,23^ The TLR4/MyD88-mediated NF-κB pathway may also interact with components of inflammation in a TXNIP-associated manner.^24^

Inflammation is an important component underlying the pathogenesis of prediabetes and T2D. IRAK-M may mediate glucose homeostasis, directly or indirectly. IRAK-M-deficient mice are more prone to the development of type 1 diabetes (T1D) and glucose intolerance.^25^ In addition, IRAK-M was shown to be involved in obesity-induced metabolic inflammation^26^ and atherogenesis.^27^ IRAK-M inhibits IRAK-1-mediated production of proinflammatory cytokines by preventing the phosphorylation of IRAK-1 and dissociation of phosphorylated IRAK-1 from the MyD88 complex, and the deletion of IRAK-1 improves muscle insulin sensitivity.^28^ TXNIP also regulates glucose and lipid metabolism by mediating β-cell function.^29^ As such, the downstream regulators of TLR signaling IRAK-M and TXNIP are suggested to be involved in T2D.

There are limited studies on the changes in IRAK-M levels in patients with prediabetes, and its relationship with related metabolic indexes, including indicators of β-cell function and insulin resistance, is unclear. In addition, although TXNIP was suggested to be highly increased in patients with T2D, it is unclear whether TXNIP is increased in the prediabetes stages. To investigate the association of IRAK-M and TXNIP changes with the development of prediabetes and T2D and assess the use of IRAK-M and TXNIP as additional indicators of prediabetes and T2D, we measured IRAK-M and TXNIP in patients with various fasting plasma glucose (FPG) levels and determined the cutoff values of IRAK-M and TXNIP for prediabetes and T2D with receiver operating characteristic curve (ROC) analysis.

## Materials and methods

### Study design and patients

In this cross-sectional study, consecutive subjects undergoing routine physical health examination in the Second Affiliated Hospital of Ningxia Medical University, China, between August 2020 and November 2020 were enrolled. Patients were excluded if they 1) were previously diagnosed with prediabetes, T1D, or T2D; (2) had acute or chronic infections; (3) had autoimmune diseases; (4) had a history of cardiovascular or cerebrovascular diseases; (5) had chronic kidney disease, liver disease, blood disease, or cancer; (6) had thyroid dysfunction; or (7) had alcohol or drug abuse. Women who were taking hormone replacement therapy were also excluded. Included subjects were classified into four groups according to their FPG values: 1) normal control (NGT), FPG < 5.6 mmol/L; 2) prediabetes A (PD-A), 5.6 mmol/L ≤ FPG <6.1 mmol/L; 3) prediabetes B (PD-B), 6.1 mmol/L ≤ FPG < 7.0 mmol/L; and 4) T2D, FPG ≥ 7.0 mmol/L. The diagnosis of prediabetes and T2D was based on the 2021 American Diabetes Association standards.^30^ All patients were newly diagnosed with prediabetes or T2D, and none had received diabetes medications or dietary supplement interventions.

This study conformed to the principles of the Declaration of Helsinki and was approved by the Ethics Committee of The First People’s Hospital of Yinchuan (No. 2021029). Written informed consent was provided by all subjects before they participated in the study.

### Data collection

Family history of T2D, personal history of complications, and individual past medical history were obtained with a unified questionnaire. Baseline characteristics, including systolic blood pressure (SBP), diastolic blood pressure (DBP), height, weight, waist circumference (WC), hip circumference, body mass index (BMI), and waist-to-hip ratio (WHR), were measured. Peripheral venous blood was collected after an 8- to 12-hour fast for laboratory tests, which included FPG, total cholesterol (TC), low-density lipoprotein (LDL), high-density lipoprotein (HDL), triglycerides (TG), alanine aminotransferase (ALT), aspartate aminotransferase (AST), creatinine (Cr), and uric acid (UA) (AU5821 automatic biochemical analyzer, Beckman Coulter, Brea, California, USA). Fasting insulin (FINS) and glycosylated hemoglobin (HbA1c) were detected by an enzyme-linked immunosorbent assay (ELISA) with a fluorescence microplate reader (Promega-GloMax, Madison, WI, USA), and the concentrations of IRAK-M and TXNIP were measured with an ELISA kit (JL46021, Jianglai Biological Co., Ltd., Shanghai, China). Homeostasis model assessment of insulin resistance (HOMA-IR) and beta-cell function (HOMA-β) were calculated as HOMA-IR = FPG × FINS/22 and HOMA-β = 20 × FINS/(FPG−3.5). The estimated glomerular filtration rate (eGFR) was calculated with the Chronic Kidney Disease Epidemiology Collaboration (CKD-EPI) equation.^31^

### Statistical analysis

Based on a preliminary estimation of a ∼15% incidence of diabetes and 40% incidence of prediabetes in a local community, a power analysis suggested a sample size of at least 370 participants would be necessary, with a power of 0.8 and a type I error (α) of 0.05. Data are expressed as a number (percentage) or mean ± standard deviation. Comparisons among different groups were performed by the one-way analysis of variance, and comparisons between two groups were performed with student’s t-test. Pearson’s correlation test and multivariate stepwise linear regression analysis were used to assess the correlation and impact between parameters, respectively. Logistic regression analysis was used to assess the odds ratio of IRAK-M and TXNIP in predicting prediabetes and T2D. A ROC curve was used to calculate the specificity and sensitivity of IRAK-M and TXNIP for the diagnosis of prediabetes and T2D. All data analyses were conducted using GraphPad Prism 8.0 software (GraphPad Software, La Jolla, CA, USA) and IBM SPSS Statistics for Windows, Version 25.0 software (IBM Corp., Armonk, NY, USA). P < 0.05 was considered to indicate a statistically significant difference.

## Results

### Demographic characteristics

Four hundred thirty subjects, including 281 men and 149 women, were included in this study and classified into the NGT (n = 119), PD-A (n = 75), PD-B (n = 123), and T2D (n = 113) groups according to their FPG. Among all included subjects, 50% had hypertension, and 30% were receiving medications (angiotensin-converting enzyme inhibitors, calcium channel blockers, or other antihypertensive drugs). The mean age, sex, and proportion of patients with hypertension were similar between the four groups (Table 1). However, there were significant differences in SBP, DBP, BMI, WC, WHR, TG, LDL, ALT, AST, HbA1c, FPG, FINS, HOMA-IR, and HOMA-β between the four groups (all p < 0.05, Table 1).

**Table 1.** Main metabolic indexes among groups.

	NGT (n = 119)	PD-A (n = 75)	PD-B (n = 123)	T2D (n = 113)	p-value
Age (years)	45.29 ± 8.93	45.39 ± 10.00	46.55 ± 7.74	46.57 ± 8.21	0.535
Men, n (%)	80 (67.23)	48 (64.00)	80 (65.04)	73 (64.60)	0.968
SBP (mmHg)	125.86 ± 14.08	135.99 ± 17.82	136.07 ± 19.11	137.00 ± 19.43	<0.001*
DBP (mmHg)	79.64 ± 11.21	85.89 ± 12.66	86.54 ± 12.66	85.19 ± 11.76	<0.001*
BMI (kg/m^2^)	24.55 ± 3.66	25.94 ± 3.23	26.35 ± 4.23	27.39 ± 8.7	0.001*
WC (cm)	81.96 ± 10.64	88.61 ± 10.50	88.77 ± 10.32	89.79 ± 9.02	<0.001*
WHR (cm/cm)	0.84 ± 0.07	0.89 ± 0.07	0.88 ± 0.10	0.90 ± 0.06	<0.001*
Hypertension, n (%)	63 (52.90)	36 (48.00)	63 (51.20)	53 (46.90)	0.792
Laboratory index					
FPG (mmol/L)	5.02 ± 0.35	5.78 ± 0.13	6.45 ± 0.24	9.36 ± 2.50	<0.001*
HbA1c (ng/mL)	165.06 ± 23.17	188.78 ± 23.54	221.06 ± 23.54	246.67 ± 23.70	<0.001*
FINS (mIU/L)	4.96 ± 0.54	5.51 ± 0.66	6.00 ± 0.48	6.76 ± 0.50	<0.001*
HOMA-IR	1.11 ± 0.15	1.42 ± 0.17	1.72 ± 0.15	2.82 ± 0.79	<0.001*
HOMA-β	69.26 ± 19.26	48.61 ± 6.27	41.01 ± 4.72	26.56 ± 8.98	<0.001*
TG (mmol/L)	1.84 ± 1.06	2.27 ± 1.50	2.48 ± 1.40	2.61 ± 1.60	<0.001*
TC (mmol/L)	4.82 ± 0.87	5.07 ± 1.15	5.18 ± 1.23	5.09 ± 0.94	0.057
HDL (mmol/L)	1.34 ± 0.24	1.35 ± 0.24	1.31 ± 0.26	1.29 ± 0.24	0.242
LDL (mmol/L)	2.64 ± 0.57	2.91 ± 0.70	3.00 ± 0.88	2.90 ± 0.61	0.001*
ALT (µmol/L)	21.97 ± 12.60	29.54 ± 21.20	36.11 ± 26.34	34.26 ± 28.65	<0.001*
AST (µmol/L)	25.49 ± 6.56	27.54 ± 8.68	32.84 ± 20.97	28.08 ± 13.13	0.001*
Cr (µmol/L)	67.91 ± 11.89	69.01 ± 11.57	66.79 ± 12.57	65.34 ± 15.87	0.268
eGFR (mL/min/1.73 m^2^)	103.64 ± 11.06	100.55 ± 10.54	102.89 ± 12.22	99.25 ± 13.32	0.350
UA (mmol/L)	338.08 ± 99.26	371.45 ± 85.68	372.58 ± 98.79	328.51 ± 97.89	0.001*
Biomarker					
IRAK-M (ng/mL)	4.74 ± 0.93	3.31 ± 0.77	3.11 ± 1.10	2.27 ± 0.67	<0.001*
TXNIP (ng/mL)	2.92 ± 0.73	4.32 ± 0.97	4.77 ± 1.18	6.33 ± 1.06	<0.001*

*p < 0.05.

NGT, normal control; PD-A, prediabetes A; PD-B, prediabetes B; T2D, type 2 diabetes; SBP, systolic blood pressure; DBP, diastolic blood pressure; BMI, body mass index; WC, waist circumference; WHR, waist-to-hip ratio; TG, triglycerides; TC, total cholesterol; HDL, high-density lipoprotein; LDL, low-density lipoprotein; ALT, alanine aminotransferase; AST, aspartate aminotransferase; Cr, creatinine; eGFR, estimated glomerular filtration rate; UA, uric acid; HbA1c, glycosylated hemoglobin; FPG, fasting plasma glucose; FINS, fasting insulin; HOMA-IR, homeostasis model assessment of insulin resistance; HOMA-β, homeostasis model assessment of β-cell function; IRAK-M, interleukin-1 receptor-associated kinase M; TXNIP, thioredoxin-interacting protein.

In addition, SBP, DBP, BMI, WC, WHR, TG, LDL, ALT, AST, HbA1c, FPG, FINS, and HOMA-IR increased significantly with higher FPG values, whereas HOMA-β significantly decreased (all p < 0.05, Table 1). LDL, ALT, AST, UA, and eGFR were not linearly associated with the FPG level; they were highest in the PD-B group but lowest in the T2D group (Table 1). In the comparison of these four groups, there was an increasing trend in the levels of FPG, HbA1c, FINS, HOMA-IR, and TG and a decreasing trend in HOMA-β levels (Table 1).

### IRAK-M and TXNIP are associated with the severity of diabetes

**Figure 1.**
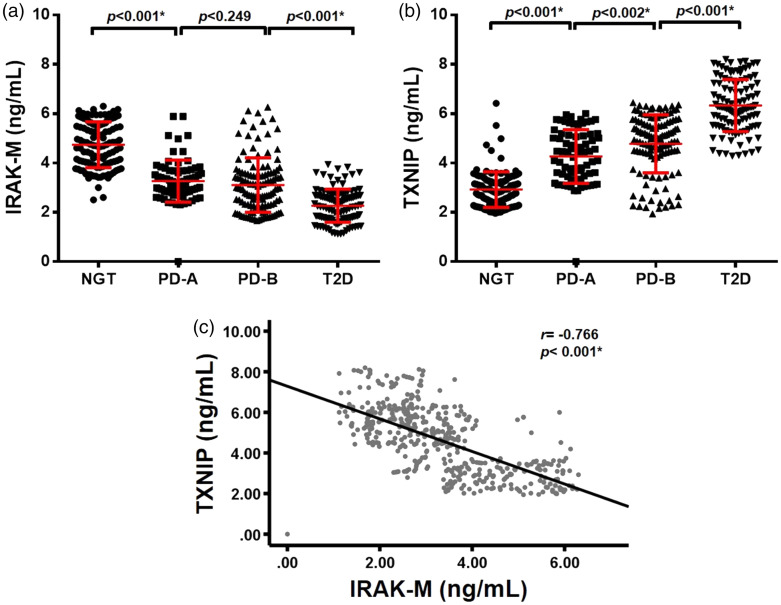
Serum IRAK-M and TXNIP levels in the four groups. Comparison of IRAK-M (a) or TXNIP levels (b) in NGT, PD-A, PD-B, and T2D groups. The red lines represent the mean ± standard deviation and (c) The correlation between IRAK-M and TXNIP was assessed by Pearson correlation analysis, *p < 0.05. IRAK-M, interleukin-1 receptor-associated kinase M; TXNIP, thioredoxin-interacting protein; NGT, normal control; PD-A, prediabetes A; PD-B, prediabetes B; T2D, type 2 diabetes.

There were highly significant differences in the levels of IRAK-M and TXNIP between these four groups (all p < 0.001, Table 1). Compared with that in the NGT group, the IRAK-M level was significantly decreased in PD-A, PD-B, or T2D groups, whereas the TXNIP level was significantly increased in these three groups (all p < 0.05, Figure 1a and 1b). There was a decreased trend in IRAK-M levels and an increased trend in TXNIP levels with the severity of diabetes. Moreover, the IRAK-M level was negatively correlated with the TXNIP level in all subjects (r = −0.766, p < 0.001, Figure 1c).

### Association of metabolic parameters with IRAK-M

By Pearson correlation analysis, IRAK-M was negatively correlated with FPG, FINS, HbA1c, and HOMA-IR and positively correlated with HOMA-β (all p < 0.001, Figure S1). Subsequent multiple stepwise linear regression analysis showed that the IRAK-M level was significantly associated with TXNIP, HbA1c, TG, HOMA-β, FINS, and UA in all subjects (all p < 0.05, Table 2). TXNIP was significantly associated with HbA1c, TG, FINS, HOMA-IR, IRAK-M, and AST in these subjects (all p < 0.05, Table 2).

**Table 2.** Multivariate stepwise linear regression analysis.

	IRAK-M		TXNIP	
Predictors	*β*	p*-*value	*β*	p*-*value
TXNIP	−0.301	<0.001*	–	–
HbA1c	−0.254	<0.001*	−0.363	<0.001*
TG	0.210	<0.001*	0.120	<0.001*
HOMA-β	0.211	<0.001*	–	–
FINS	−0.110	0.005*	0.112	0.020*
UA	−0.074	0.021*	–	–
HOMA-IR	–	–	0.178	<0.001*
IRAK-M	–	–	−0.257	<0.001*
AST	–	–	−0.071	0.016*

*p < 0.05.

TG, triglycerides; AST, aspartate aminotransferase; UA, uric acid; HbA1c, glycosylated hemoglobin; FINS, fasting insulin; HOMA-IR, homeostasis model assessment of insulin resistance; HOMA-β, homeostasis model assessment of β-cell function; IRAK-M, interleukin-1 receptor-associated kinase M; TXNIP, thioredoxin-interacting protein.

### IRAK-M or TXNIP is an independent indicator of diabetes

**Figure 2.**
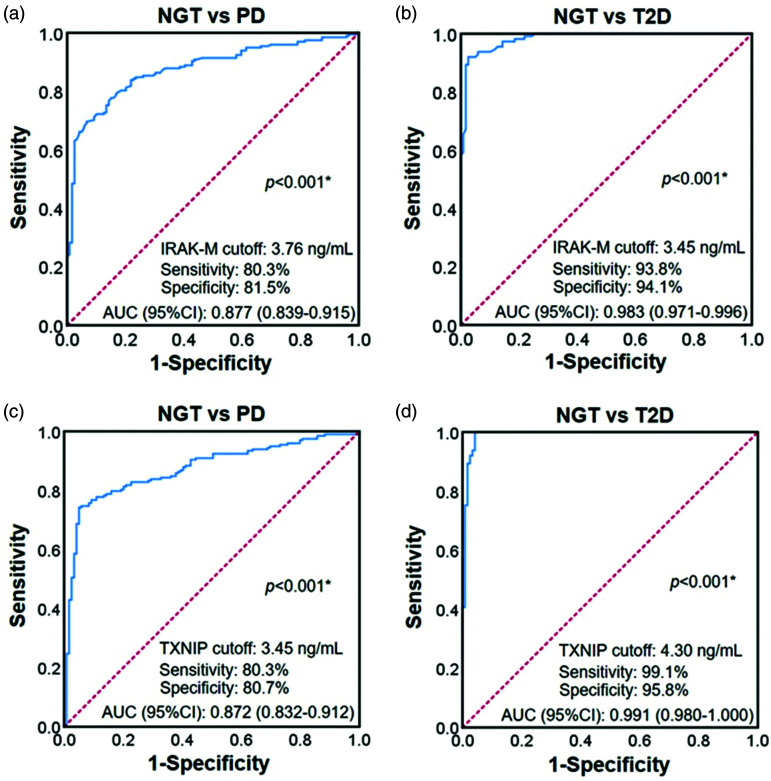
ROC curve for IRAK-M and TXNIP for the prediction of prediabetes or T2D. The ROC analysis showed that the optimal cutoff values of IRAK-M for prediabetes (a) and T2D (b) were 3.76 ng/mL and 3.45 ng/mL, respectively. The AUCs for prediabetes and T2D were 0.877 and 0.983, respectively. The ROC analysis showed that the optimal cutoff values of TXNIP for prediabetes (c) and T2D (d) were 3.45 ng/mL and 4.30 ng/mL, respectively. The AUCs for prediabetes and T2D were 0.872 and 0.991, respectively. *p < 0.05. ROC, receiver operating characteristic; IRAK-M, interleukin-1 receptor-associated kinase M; TXNIP, thioredoxin-interacting protein; T2D, type 2 diabetes; AUC, area under the curve.

In the logistic regression analysis with or without adjustments for confounding factors, subjects with higher IRAK-M levels had a lower risk of prediabetes and T2D (all p < 0.05, Table 3). In addition, subjects with higher TXNIP levels had a higher risk of prediabetes and T2D (all p < 0.05, Table 3). Furthermore, we performed ROC curve analysis of IRAK-M or TXNIP for prediabetes and T2D. As shown in Figure 2a, the diagnostic cutoff value of the IRAK-M level for prediabetes was 3.76 ng/mL, yielding an area under the curve (AUC) of 0.877, sensitivity of 80.3%, and specificity of 81.5% (p < 0.001). The diagnostic cutoff value of the IRAK-M level for T2D was 3.45 ng/mL, yielding an AUC of 0.983, sensitivity of 93.8%, and specificity of 94.1% (p < 0.001, Figure 2b). In addition, the diagnostic cutoff value of the TXNIP level for prediabetes was 3.45 ng/mL, yielding an AUC of 0.872, sensitivity of 80.3%, and specificity of 80.7% (p < 0.001, Figure 2c). The diagnostic cutoff value of the TXNIP level for T2D was 4.30 ng/mL, yielding an AUC of 0.991, sensitivity of 99.1%, and specificity of 95.8% (p < 0.001, Figure 2d).

**Table 3.** Logistic regression analysis of predictors and diabetes.

		PD-A		PD-B		T2D	
Model	NGT	aOR (95% CI)	p*-*value	aOR (95% CI)	p*-*value	aOR (95% CI)	p*-*value
IRAK-M							
Unadjusted	Ref.	0.15 (0.09,0.26)	<0.001*	0.28 (0.18,0.35)	<0.001*	0.02 (0.01,0.06)	<0.001*
Model 1	Ref.	0.16 (0.08,0.27)	<0.001*	0.27 (0.19,0.39)	<0.001*	0.01 (0.01,0.04)	<0.001*
Model 2	Ref.	0.15 (0.07,0.31)	<0.001*	0.46 (0.28,0.77)	0.003*	0.05 (0.01,0.50)	0.010*
TXNIP							
Unadjusted	Ref.	5.02 (3.12,8.07)	<0.001*	4.83 (3.36,6.94)	<0.001*	22.16 (8.40,58.49)	<0.001*
Model 1	Ref.	4.46 (2.75,7.23)	<0.001*	4.89 (3.25,7.35)	<0.001*	51.75 (9.43,283.99)	<0.001*
Model 2	Ref.	4.35 (2.22,8.53)	<0.001*	2.94 (1.76,4.90)	<0.003*	8.64 (1.31,56.90)	0.025*

Logistic model 1: adjustment for age, sex, SBP, DBP, BMI, WC, WHR, and hypertension.

Logistic model 2: adjustment for age, sex, SBP, DBP, BMI, WC, WHR, hypertension, TG, LDL, ALT, AST, UA, and IRAK-M/or TXNIP.

*p < 0.05.

NGT, normal control; PD-A, prediabetes A; PD-B, prediabetes B; T2D, type 2 diabetes; aOR, adjusted odds ratio; CI, confidence interval; SBP, systolic blood pressure; DBP, diastolic blood pressure; BMI, body mass index; WC, waist circumference; WHR, waist-to-hip ratio; TG, triglycerides; LDL, low-density lipoprotein; ALT, alanine aminotransferase; AST, aspartate aminotransferase; UA, uric acid; IRAK-M, interleukin-1 receptor-associated kinase M; TXNIP, thioredoxin-interacting protein.

After stratifying by IRAK-M (<3.76 and ≥3.76 ng/mL) according to the data shown in Figure 2a, we further assessed whether IRAK is associated with TXNIP-related diabetes. As shown in Table S1, logistic regression analysis revealed that patients with a higher TXNIP level had a greater risk of PD-A, PD-B, or T2D in the subgroup with low IRAK-M (<3.76 ng/mL), before and after adjusting for confounding factors (all p < 0.05). However, those with a higher TXNIP level only had a greater risk of PD-A in the subgroup with high IRAK-M (≥3.76 ng/mL), before and after adjusting for confounding factors (all p < 0.05).

## Discussion

In this study, we found that the plasma level of IRAK-M was significantly lower in patients with newly diagnosed prediabetes or T2D. IRAK-M was significantly positively correlated with HOMA-β and significantly negatively correlated with FPG, FINS, HbA1c, and HOMA-IR. These results suggest that decreased IRAK-M is associated with prediabetes and T2D. Moreover, the plasma level of TXNIP was significantly higher in patients with newly diagnosed prediabetes or T2D. TXNIP was also negatively correlated with IRAK-M, suggesting that increased TXNIP is associated with prediabetes and T2D. These results revealed a reduced trend in IRAK-M and an elevated trend in TXNIP with increasing FPG levels. Logistic regression analysis indicated that IRAK-M is an independent negative predictor of prediabetes and T2D, while TXNIP is an independent positive predictor of prediabetes and T2D. The high AUC values for IRAK-M (0.877) and TXNIP (0.872) in prediabetes and IRAK-M (0.983) and TXNIP (0.991) in T2D further suggested that IRAK-M and TXNIP are reliable markers of early T2D development. T2D is a metabolic disorder associated with uncontrolled hyperglycemia and chronic inflammation that can lead to islet β-cell injuries. Under normal physiological conditions, insulin secreted by β-cells lowers blood glucose by activating insulin receptors on the surface of various cells, including adipocytes, cardiocytes, and hepatocytes, and increases glucose uptake. However, insulin resistance affects the metabolism of several cell types, resulting in endoplasmic reticulum stress, oxidative stress, lipid homeostasis dysregulation, and mitochondrial dysfunction, which further leads to the development of T2D and its complications.^32,33^ The development of T2D and its complications is also associated with abnormal immune responses and chronic inflammation,^34^ and innate immunity is highly associated with T2D.^35^ The functions of innate immune cells involved in T2D are mainly mediated via TLRs, particularly TLR2 and TLR4.^36^ TLRs recognize exogenous and endogenous antigens and produce pro-inflammatory cytokines and chemokines, resulting in chronic inflammation that may damage β-cells and lead to T2D.^1,36^ Several molecules are involved in TLR signaling.^37^ Among them, IRAK-M mainly restricted to monocytes/macrophages is a negative regulator of TLR signaling pathways.^21^ IARK-M is induced upon TLR stimulation and then prevents the dissociation of IRAK-1 and IRAK-4 from MyD88 and the formation of the IRAK-1-TRAF-6 complex.^21^ A previous study with nonobese diabetic mice found that IRAK-M deficiency (IRAK-M^−/−^) led to the early onset and rapid progression of T1D, accompanied by more severe insulitis and elevated anti-insulin autoantibodies.^25^ In our cross-sectional study with human subjects classified according to FPG, the level of IRAK-M was significantly decreased in patients with T2D and also during the prediabetes stages (PD-A and PD-B groups). Therefore, we speculate that before T2D develops, abnormalities in TLR signaling occur in an IRAK-M-associated manner.

TXNIP is another chronic inflammation-related factor that may cause islet β-cell dysfunction.^38^ TXNIP is an activator of the NOD-, LRR- and pyrin domain-containing protein 3 (NLRP3) inflammasome, mediating oxidative stress by interacting with and inhibiting thioredoxin.^38,39^ Under high glucose conditions, the gene expression of *TXNIP* is upregulated in islet cells. In addition, reactive oxygen species (ROS) are induced under hyperglycemia, subsequently triggering the release of TXNIP from thioredoxin, activating TXNIP/NLRP3 and a series of related inflammatory reactions, and leading to β-cell apoptosis.^40–42^ Recently, NADPH oxidase 5 (NOX5) was identified as a downstream target of the TLR pathway, and NOX5-derived ROS may be modulated by IRAK.^43^ Furthermore, ROS activate NLRP3 by promoting TXNIP and NIMA-related kinase 7 interactions with NLRP3. The ROS production induced by IRAK may contribute to NLRP3 activation initiated by the activation of these kinases.^41,44,45^ In addition, TXNIP was shown to be highly positively correlated with NLRP3, caspase-1, and IL-1β, and TXNIP was abnormally expressed as the FPG level increased, suggesting that TXNIP augments the body’s inflammatory response to oxidative stress.^46^ TXNIP inhibits insulin expression and glucose uptake, and insulin resistance can lead to elevated TXNIP levels in islet β-cells.^40,47^ Accordingly, we speculate that IRAK-M/TLR-related diabetes may be associated with the ROS/TXNIP signaling pathway.

The pathogenesis of T2D involving TLR-mediated chronic inflammation is complex. In addition to IRAK-M and TXNIP suggested in the present study, other downstream mediators of TLRs (TLR2 and TLR4) may play a role. A recent study revealed the novel regulation of TLR4 in a mouse model whereby the ribosome biosynthesis protein NOC4 binds to and inhibits the endocytosis of TLR4 and blocks the TRIF pathway. This consequently inhibits the production of inflammatory cytokines, subsequently improving local and systemic inflammation and reducing insulin resistance.^48^ Another study revealed that TLR4 knockout activated TRIF/IRF3 signaling, induced inflammation, and increased the spleen index.^49^ Studies with TLR2 knockout (*Tlr2*^−/−^) mice revealed other possible mechanisms leading to diabetic and obese phenotypes. For example, the bone morphogenic protein-induced binding between TLR2 and NOX1 or NOXO1 was abrogated, and the high-fat diet-induced increases in FPG and insulin levels in wild-type mice were restored in *Tlr2* ^−/−^ mice.^50^

An association between IRAK-M reduction and the development of diabetes has also been suggested in other studies. In a study that used a dendritic cell modulator for the treatment of T1D in nonobese diabetic mice, the induction of IRAK-M was found to be accompanied by the increased efficacy of this drug (i.e., delaying the onset of insulitis and preventing the onset of hyperglycemia).^51^ Another study revealed that IRAK-M^−/−^ nonobese diabetic mice had early onset and rapid progression of T1DM and impaired glucose tolerance.^52^ However, the upregulation of IRAK-M may also imply a harmful condition. In a mouse model with hypoxia-inducible factor-1α overexpression in macrophages, a high-fat diet increased the expression of IRAK-M, leading to macrophage infiltration, inflammation, and fibrosis in adipose tissue. Moreover, global *Irak-M* deficiency was associated with reduced fibrosis and improved glucose tolerance, suggesting an IRAK-M-dependent mechanism in obesity-related adipose tissue dysfunction.^53^

Previously, we reported changes in the level of various metabolic parameters and several physical characteristics with increasing FBG levels.^46^ Here, with newly enrolled subjects, we showed similar trends in SBP, DBP, BMI, WC, WHR, TG, LDL, FPG, FINS, HOMA-IR, and HOMA-β. In addition, to further classify the subjects with prediabetes into the PD-A (5.6 mmol/L ≤ FPG < 6.1 mmol/L) and PD-B (6.1 mmol/L ≤ FPG < 7.0 mmol/L) groups, we examined HbA1c, Cr, ALT, and AST in this study. When comparing the PD-A group with the PD-B group, only HbA1c, FINS, HOMA-IR, and HOMA-β showed significant differences, and there were no significant differences in blood pressure, body weight, BMI, WC, WHR, TG, TC, HDL, LDL, ALT, AST, UA, and Cr. These results suggested that during the early development of T2D, only characteristics directly related to the plasma glucose level were affected. The decreasing HOMA-β values with increasing FPG levels indicate more severe β-cell dysfunction, as expected in people with T2D. However, the values of ALT, AST, and UA, which are indicators of liver dysfunction and cardiovascular disease, were the highest in the PD-B group but lowest in the T2D group. The liver is one of the main organs responsible for glycogen synthesis and glucose homeostasis maintenance, and elevated levels of liver enzymes are clinical indicators of liver dysfunction. The plasma levels of liver enzymes have been shown to be positively correlated with FPG, and patients with T2D often have liver dysfunction.^54–56^ Our results showed that the levels of ALT and AST were highest in the PD-B group rather than in the T2D group; therefore, liver function might have deteriorated before the FPG level reached the diagnostic threshold for T2D. Similarly, the level of UA increased as the FPG level increased but was highest in the PD-B group and decreased in the T2D group. UA has been suggested to be a risk factor for cardiovascular disease^57^ and dynamically associated with the level of HbA1c. In addition, the UA level might be higher in patients with prediabetes than in those with T2D.^58^ Our results suggested that liver and cardiovascular diseases might have developed during the early stages of T2D.

Glucose homeostasis or diabetes is usually assessed or identified with the surrogate biomarkers FPG and HbA1c in clinical practice.^59,60^ However, as suggested by the Diabetes Control and Complications Trial,^61^ using a single measurement for FPG may be misleading. Previous studies have shown that for patients with chronic kidney disease or microangiopathy or elderly patients, glycated albumin is a more reliable indicator of the glycemic status than HbA1c because of the reduced survival of erythrocytes and consequent decrease in the time available for glucose to attach to hemoglobin.^60,62^ In addition, because subjects with impaired FPG usually have other metabolic syndromes, cardiovascular disease, or cerebrovascular disease, additional surrogate biomarkers, including galectin-3, high sensitivity cardiac troponin I, hyperhomocysteinemia, and heart-type fatty acid binding protein, may be considered for a more comprehensive interpretation of the underlying pathological mechanisms.^59,63–65^

This study has several limitations. First, the unequal numbers of subjects among the four groups may lead to heteroscedasticity during analysis. Second, the physiological effects of IRAK-M and TXNIP are exerted intracellularly, but only IRAK-M and TXNIP levels outside the cells were estimated. Third, the mechanism of T2D development is complicated; factors other than IRAK-M, TXNIP, and metabolic indicators revealed in this study may also play a role. Fourth, albuminuria values were not examined in the present study, and a possible effect of early kidney dysfunction on IRAK-M and TXNIP changes cannot be ruled out. Further studies are warranted to elucidate these issues.

In conclusion, our results provide evidence that IRAK-M is independently and positively associated with prediabetes and T2D, while TXNIP is independently and negatively associated with prediabetes and T2D. IRAK-M and TXNIP showed high AUC values for patients with prediabetes. Therefore, the combination of low IRAK-M and high TXNIP might be diagnostic for prediabetes.

## Supplemental Material

sj-jpg-1-imr-10.1177_03000605221111275 - Supplemental material for Changes in plasma IRAK-M in patients with prediabetes and its relationship with related metabolic indexes: a cross-sectional study Click here for additional data file. Supplemental material, sj-jpg-1-imr-10.1177_03000605221111275 for Changes in plasma IRAK-M in patients with prediabetes and its relationship with related metabolic indexes: a cross-sectional study by Xiaomin Xie, Guirong Bai, Li Zhang, Huili Liu, Dan Qiang and Ling Li in Journal of International Medical Research

sj-pdf-2-imr-10.1177_03000605221111275 - Supplemental material for Changes in plasma IRAK-M in patients with prediabetes and its relationship with related metabolic indexes: a cross-sectional study Click here for additional data file. Supplemental material, sj-pdf-2-imr-10.1177_03000605221111275 for Changes in plasma IRAK-M in patients with prediabetes and its relationship with related metabolic indexes: a cross-sectional study by Xiaomin Xie, Guirong Bai, Li Zhang, Huili Liu, Dan Qiang and Ling Li in Journal of International Medical Research
