# Supplementary figures and images for: Changes in plasma IRAK-M in patients with prediabetes and its relationship with related metabolic indexes: a cross-sectional study
Source: J Int Med Res. 2022 Aug 30;50(8):03000605221111275. doi: 10.1177/03000605221111275 (PMC9437484; doi:10.1177/03000605221111275)

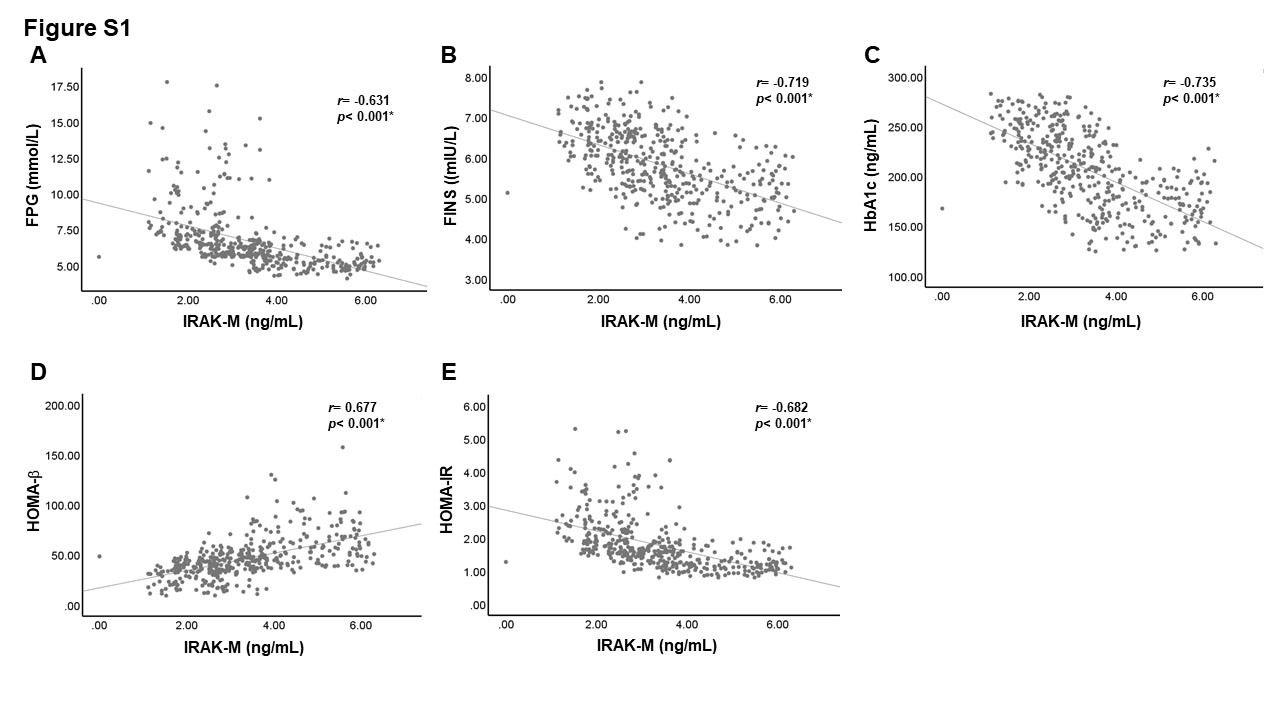

Supplement: sj-jpg-1-imr-10.1177_03000605221111275 - Supplemental material for Changes in plasma IRAK-M in patients with prediabetes and its relationship with related metabolic indexes: a cross-sectional study [file sj-jpg-1-imr-10.1177_03000605221111275.jpg]
