# Supplementary material for: Changes in plasma IRAK-M in patients with prediabetes and its relationship with related metabolic indexes: a cross-sectional study
Source: J Int Med Res. 2022 Aug 30;50(8):03000605221111275. doi: 10.1177/03000605221111275 (PMC9437484; doi:10.1177/03000605221111275)
Supplement: sj-pdf-2-imr-10.1177_03000605221111275 - Supplemental material for Changes in plasma IRAK-M in patients with prediabetes and its relationship with related metabolic indexes: a cross-sectional study [file sj-pdf-2-imr-10.1177_03000605221111275.pdf]

Table S1. Logistic regression analysis of TXNIP and diabetes after classifying by IRAK-M level.

	Model	NGT	PD-A		PD-B		T2D	
			aOR (95% CI)	***p*-value**	***aOR (95% CI)***	***p*-value**	***aOR (95% CI)***	***p*-value**
**IRAK-M**								
<3.76 ng/mL	Unadjusted	Ref.	2.61(1.41,4.86)	0.002*	5.86(3.12,10.98)	<0.001*	10.29(3.93,26.99)	<0.001*
	Model 1	Ref.	3.10(1.54,6.23)	0.001*	6.70(3.03,14.83)	<0.001*	16.01(2.02,50.12)	0.021*
	Model 2	Ref.	3.55(1.47,8.55)	0.005*	6.38(2.55,15.93)	<0.001*	17.63(2.46,83.26)	0.036*
≧3.76 ng/mL	Unadjusted	Ref.	8.78(3.63,21.25)	<0.001*	1.58(0.84,2.97)	0.160	-	-
	Model 1	Ref.	22.15(3.47,67.02)	0.008*	2.13(0.94,4.81)	0.069	-	-
	Model 2	Ref.	25.86(4.02,71.20)	0.012*	2.27(0.92,5.57)	0.074	-	-

NGT, normal control; PD-A, prediabetes A; PD-B, prediabetes B; T2D, type 2 diabetes. *$p< 0.05$.

Logistic model 1: adjustment for age, gender, SBP, DBP, BMI, WC, WHR, and hypertension.

Logistic model 2: adjustment for age, gender, SBP, DBP, BMI, WC, WHR, hypertension, TG, LDL, ALT, AST, UA, and IRAK-M/or TXNIP.
